# Supplementary material for: Heat adaptation of phage T7 under an extended genetic code
Source: Virus Evol. 2021 Dec 1;7(2):veab100. doi: 10.1093/ve/veab100 (PMC8923235; doi:10.1093/ve/veab100)
Supplement: veab100_Supp [file veab100_supp.zip › Sequencing_Analysis.zip!/Sequencing_Analysis/A2/evidence/SNP_8.html]

BRESEQ :: Evidence


| Predicted mutation | | | | | | |
| --- | --- | --- | --- | --- | --- | --- |
| evidence | seq id | position | mutation | annotation | gene | description |
| RA | NC\_001604.1 | 24,238 | A→G | intergenic (–/–) | – / – | –/– |

| Read alignment evidence... | | | | | | | | | | | |
| --- | --- | --- | --- | --- | --- | --- | --- | --- | --- | --- | --- |
|  | seq id | position | | ref | new | freq | score (cons/poly) | reads | annotation | genes | product |
| \* | NC\_001604.1 | 24,238 | 0 | A | G | 98.6% | 1109.8 / NA | 371 | intergenic (–/–) | *–/–* | –/– |
| Reads supporting (aligned to +/- strand):  **ref** base A (1/2);  **major** base G (199/164);  **minor** base T (3/2);  **total** (203/168) | | | | | | | | | | | |
| Fisher's exact test for biased strand distribution *p*-value = 1.00e+00 | | | | | | | | | | | |
| Kolmogorov-Smirnov test that lower quality scores support variant *p*-value = 9.99e-01 | | | | | | | | | | | |

Only 100 of 371 total aligned reads displayed.  

`GCAATGGGCCACGGTGGTCTTCGCCCAGAAGCTGCTGGTGCAGTGGTTTTCAAAGTGGAGTAATGCTGGGGGTGGCCTCAACGGTCGCTGCTAGTCCCGAAGAGGCGAGTGTTACTTCAACAGAAGAAACCTTAACGCCAGCACAGGAGGCCGCACGCACCCGCGCTGCTAACAAAGCCCGAAAGGAAGCTGAGTTGGCTGCTGCCACCGCTGAGCAATAACTAGCATAACCCCTTGGGGCCTCTAAACGGGTCTTGAGGGGTTTTTTGCTGAAAGGAGGAACTATATGCGCTCATACGATATGAACGTTGAGACTGCCGCTGAGTTATCAGCTGTGAACGACATTCTGGCGTCTATCGGTGAACCTCCGGTATCAACGCTGGAAGGTGACGCTAACGCAGATGCAGCGAACGCTCGGCGTATTCTCAACAAGATTAACCGACAGATTCAATCTCGTGGATGGACGTTCAACATTGAGGAAGGCATAACGCTACTACCTGATGTTTACTCCAACCTGATTGTATACAGTGACGACTATTTATCCCTAATGTCTACTTCCGGTCAATCCATCTACGTTAACCGAGGTGGCTATGTG  >  NC_001604.1/23942‑24536`  

                                                                                                                                                                                                                                                                                                        `|`                                                                                                                                                                                                                                                                                                            
`gCAATGGGCCACGGTGGTCTTCGCCCAGAAGCTGCTGGTGCAGTGGTTTTCAAAGTGGAGTAATGCTGGGGGTGGCCTCAACGGTCGCTGCTAGTCCCGAAGAGGCGAGTGTTACTTCAACAGAAGAAACCTTAACGCCAGCACAGGAGGACGCACGCACCCGCGCTGCTAACAAAGCCCGAAAGGAAGCTGAGTTGGCTGCTGCCACCGCTGAGCAATCACTAGCATAA‑CCCTTGGGGCCTCTAAACGGGTCTTGAGGGGTTTTTTGCTGAAAGGAGGAACTATATGCGCTCATGCAata                                                                                                                                                                                                                                                                                                       <  1:260644/301‑1 (MQ=255)`  
                                `gacaggggCAGTGGTTTTCAAAGTGGAGTAATGCTGTGGGTGGCCTCAACGGTCGCTGCTAGTCCCGAAGAGGCGAGGGTTACTTCAACAGAAGAAACCTTAACGCCAGCACAGGAGGCCGCACGCACCCGCGCTGCTAACAAAGCCCGAAAGGAAGCTGAGTTGGCTGCTGCCACCGCTGAGCAATCACTAGCATAACCCCTTGGGGCCTCTAAACGTGTCTTGAGGGGTTTTTTGCTGAAAGGAGGAACTATATGCGCTCATGCGATATGAACGTTGAGACTGCCGCTGAGTTATCAGc                                                                                                                                                                                                                                                                        <  1:704712/294‑1 (MQ=255)`  
                                `aacaggTGCAGTGGTTTTCAAAGTGGAGTAATGCTGGGGGTGGCCTCAACGGTCGCTGCTAGTCCCGAAGAGGCGAGTGTTACTTCAACAGAAGAAACCTTAACGCCAGCACAGGAGGCCGCACGCACCCGCGCTGCTAACAAAGCCCGAAAGGAAGCTGAGTTGGCTGCTGCCACCGCTGAGCAATCACTAGCATAACCCATTGGGGACTCTAAACGGGGCTTGAGGGGTTTTTTGCTGAAAGGAGGAACTATATGCGCTCATGAGATATGAACGTTGAGACTGCCGCTGAGTTATCAGc                                                                                                                                                                                                                                                                        <  1:167473/297‑1 (MQ=255)`  
                                          `gttgTTTTCAAAGTGGAGTAATGCTGGGGGTGGCCTCAACGGTCGCTGCTAGTCCCGAAGAGGCGAGTGTTACTTCAACAGAAGAAACCTTAACGCCAGCACAGGAGGCCGCACGCACCCGCGCTGCTAACAAAGCCCGAAAGGAAGCTGAGTTGGCTGCTGCCACCGCTGAGCAATCACTAGCATAACCCCTTGGGGCCTCTAAACGGGTCTTGAGGGGTTTTTTGCTGAAAGGAGGAACTATATGCGCTCATGCGATATGAACGTTGAGACTGCCGCTGAGTTATCAGCTGTGAACGAc                                                                                                                                                                                                                                                              >  1:314396/4‑301 (MQ=255)`  
                                                   `agacaggAATAATGCTGGGGGTGGCCTCAACGGTCGCTGCTAGTCCCGAAGAGGCGAGTGTTACTTCAACAGAAGAAACCTTAACGCCAGCACAGGAGGCCGCACGCACCCGCGCTGCTAACAAAGCCCGAAAGGAAGCTGAGTTGGCTGCTGCCACCGCTGAGCAATCACTAGCATAACCCCTTGGGGCCTCTAAACGGGTCTTGAGCGGTTTTTTGCTGAAAGGAGGAACTATATGCGCTCATGCGATATGAACGTTGAGACTGCCGCTGAGTTATCAGCTGTGAACGACATTCTGGCg                                                                                                                                                                                                                                                     <  1:455702/296‑1 (MQ=255)`  
                                                         `gAGTAATGCTGGGGGTGGCCTCAACGGTCGCTGCTAGTCCCGAAGAGGCGAGTGTTACTTCAACAGAAGAAACCTTAACGCCAGCACAGGAGGCCGCACGCACCCGCGCTGCTAACAAAGCCCGAAAGGAAGCTGAGTTGGCTGCTGCCACCGCTGAGCAATCACTAGCATAACCCCTTGGGGCATCTAAACGGGTCTTGAGGGGTTTTTTGCTGAAAGGAGGAACTATATGCGATCATGCGATATGAACGTTGAGACTGCCGCTGAGTTATCAGCTGTGAAcctgtcgcttatacacatt                                                                                                                                                                                                                                               >  1:424397/1‑283 (MQ=255)`  
                                                         `gAGTAATGCTGGGGGTGGCCTCAACGGTCGCTGCTAGTCCCGAAGAGGAGAGTGTTACTTAAACAGAAGAAACCTTAACGCCAGCACAGGAGGCCGCACGCACCCGCGCTGCTAACAAAGCCCGAAAGGAAGCTGAGTTGGCTGCTGCCACCGCTGAGCAATCACTAGCATAACCCCTTGGGGCCTCTAAACGGGTCTTGAGGGGTTTTTTGCTGAAAGGAGGAACTATATGCGCTCATGCGATATGAACGTTGAGACTGCCGCTGAGTTATCAGCTGTGAACGACATTCTGGCGTCTATc                                                                                                                                                                                                                                               >  1:121194/1‑301 (MQ=255)`  
                                                           `gTAATGCTTGGGGTGGCCTCAACGGTCGCTGCTAGTCCCGAATAGGCGAGTGTTACTTCAACAGAAGAAACCTTAACTCCAGCACAGGAGGCCGCACGCACCCGCGCTTCTAACAAAGCCCGAAAGGAAGCTGAGTTGGCTGCTGCCACCGCTGAGCAATAACTAGCATAACCCCTTGGGGCCTCTAAACGGGTCTTGAGGGGTTTTTTGCTGAAAGGAGGAACTATATGCGCTCATGCGATATGAACGTTGAGACTGCCGCTGAGTTATCAGCTGTGAACGACATTCTGGCGTCTATCgg                                                                                                                                                                                                                                             >  1:526924/1‑301 (MQ=255)`  
                                                           `gTAATGCTGGGGGTGGCCTCAACGGTCGCTGCTAGTCCCGAAGAGGCGAGTGTTACTTCAACAGAAGAAACCTTAACGCCAGCACAGGAGGCCGCACGCACCCGCGCTGCTAACAAAGCCCGAAAGGAAGCTGAGTTGGCTGCTGCCACCGCTGAGCAATCACTAGCATAACCCCTTGGGGCCTCTAAACGGGTCTTGAGGGGTTTTTTGCTGAAAGGAGGAACTATATGCGCTCATGCGATATGAACGTTGAGACTGCCGCTGAGTTATCAGCTGTGAACGACATTCTGGCGTCTATCgg                                                                                                                                                                                                                                             <  1:174156/301‑1 (MQ=255)`  
                                                              `aTGCTGGGGGTGGCCTCAACGGTCGCTGCTAGTCCCGAAGAGGCGAGTGTTACTTCAACAGAAGAAACCTTAACGCCAGCACAGGAGGCCGCACGCACCCGCGCTGCTAACAAAGCCCGAAAGGAAGCTGAGTTGGCTGCTGCCACCGCTGAGCAATCACTAGCATAACCCCTTGGGGCCTCTAAACGGGTCTTGAGGGGTTTTTTGCTGAAAGGAGGAACTATATGCGCTCATGCGATATGAACGTTGAGACTGCCGCTGAGTTATCAGCTGTGAACGACATTCTGGCGTCTATCGGTGa                                                                                                                                                                                                                                          >  1:631106/1‑301 (MQ=255)`  
                                                                   `taagtataagggacaggTCGCTGCTAGTCCCGGAGAGGCGAGTGTAACTTCAACAGAAGAAACCTGAGATCCAGCACAGGAGGCAGCACGCACCCGCGCTGCTAACAAAGCCCGAAAGGAAGCTGAGTTGGCTGCTGCCACCGCTGAGCAATCACTAGCATAACCCCTTGGGGCCTCTAAACGGGTCTTGATGGGTTTTATGCTGAAAGGAGGAACTATATGCGCTCATGCGATATGAACGTTGAGACTGCCGCTGAGTTATCAGCTGTGAACGACATTCTGGCGTCTATCGGTGAACCTc                                                                                                                                                                                                                                     <  1:248304/286‑1 (MQ=255)`  
                                                                        `ggagatgtgtataagagacaggTCCCGAAGAGGCGAGTGTTACTTCAACAGAAGAAACCTTAACGCCAGCACAGGAGGCCGCACGCACCCGCGCTGCTAACAAAGCCCGAAAGGAAGCTGAGTTGGCTGCTGCCACCGCTGAGCAATCACTAGCATAACCCCTTGGGGCCTCTAAACGGGTCTTGAGGGGTTTTTTGCTGAAAGGAGGAACTATATGCGCTCATGCGATATGAACGTTGAGACTGCCGCTGAGTTATCAGCTGTGAACGACATTCTGGCGTCTATCGGTGAACCTCCGGTa                                                                                                                                                                                                                                <  1:258767/280‑1 (MQ=255)`  
                                                                         `ggCCTCCACGGTCGCTGCTAGTCCCGAAGAGGCGAGTGTTACTTCAACAGAAGAAACCTTAACGCCAGCACAGTAGGCCGCACGCACCCGCGCTGCTAACAAAGCCCGAAAGGAAGCTGAGTTGGCTGCTGCCACCGCTGAGTAATCACTAGCATAACCCCTTGGGGCCTCTAAACGGGTCTTGATGGGTTTTTTGCTGAAAGGAGGAACTATATGCGCTCATGCGATATGAACGTTGAGACTGCCGCTGAGTTATCAGCTGTGAACGACATTCTGGTGTCTATCGGTGAACCTCCAGTAt                                                                                                                                                                                                                               <  1:252147/301‑1 (MQ=255)`  
                                                                              `cAACGGTCGCTGCTAGTCCCGAAGAGGCGAGTGTTACTTCAACAGAAGAAACCTTAACGCCAGCACAGAAGGCCGCACGCACCCGCGCTGCTAACAAAGCCCGAAAGGAAGCTGAGTTGGCTGCTGCCACCGCTGAGCAATCACTAGCATAACCCCTTGGGGCCTCTAAACGGGTCTTGAGGGGTTTTTTGCTGAAAGGAGGAACTATATGCACTCATGCGATATGAACGTTGAGACTGCCGCTGAGTTATCAGCTGTGAACGACATTCTGGCGTCTATCGGTGAACCTCCGGTATCAACg                                                                                                                                                                                                                          >  1:76298/1‑301 (MQ=255)`  
                                                                                             `gTCCCGAAGAGGCGAGTGTTACTTCAACAGAAGAAACCTTAACGCCAGCACAGGAGGCCGCACGCACCCGCGCTGCTAACAAAGCCCGAAAGGAAGCTGAGTTGGCTGCTGCCACCGCTGAGCAATCACTAGCATAACCCCTTGGGGCCTCTAAACGGGTCTTGAGGGGTTTTTTGCTGAAAGGAGGAACTATATGCGCTCATGCGATATGAACGTTGAGACTGCCGCTGAGTTATCAGCTGTGAACGACATTCTGGCGTCTATCAGTAAACCTCCGGTATCAACGcctgtctcttataca                                                                                                                                                                                                           >  1:447563/1‑287 (MQ=255)`  
                                                                                                `ccGAAGAGGCGAGTGTTACTTCAACAGAAGAAACCTTAACGCCAGCACAGGAGGCCGCACGCACCCGCGCTGCTAACAAAGCCCGAAAGGAAGCTGAGTTGGCTGCTGCCACCGCTGAGCAATCACTAGCATAACCCCTTGGGGCCTCTAAACGGGTCTTGAGGGGTTTTTTGCTGAAAGGAGGAACTATATGCGCTCATGCGATATGAACGTTTAGACTGCCGCTGAGTTATCAGCTGTGAACGACATTCTGGCTTCTATCGGTTAACCTccctgtctcttatacacatctccgagcccc                                                                                                                                                                                                        >  1:83295/1‑273 (MQ=255)`  
                                                                                                   `aaGAGGCGAGTGTTACTTCAACAGAAGAAACCTTAACGCCAGCACAGGAGGCCGCACGCACCCGCGCTGCTAACAAAGCCCGAAAGGAAGCTGAGTTGGCTGCTGCCACCGCTGAGCAATCACTAGCATAACCCCTTGGGGCCTCTAAACGGGTCTTGAGGGGTTTTTTGCTGAAAGGAGGAGCTATATGCGCTCATGCGATATGAACGTTGAGACTGCCGCTGAGTTATCAGCTGTGAACGACATTCTGGCGTCTATCGGTGAACCTCCGGTATCAACGCTGGAAGGTGACGCTAACGcc                                                                                                                                                                                                     >  1:269446/1‑300 (MQ=255)`  
                                                                                                      `gagatgtgtataagagacaggaagaaACCTTAACGCCAGCACAGGAGGCCGCACGCACCCGCGCTGCTAACAAAGCCCGAAAGGAAGCTGAGTTGGCTGCTGCCACCGCTGAGCAATCACTAGCATAACCCCTTGGGGCCTCTAAACGGGTCTTGAGGGGTTTTTTGCTGAAAGGAGGAACTATATGCGCTCATGCGATATGAACGTTGAGACTGCCGCTGAGTTATCAGCTGTGAACGACATTCTGGCGTCTATCGGTGAACCTCCGGTATCAACGCTGGAATGTGACGCTAACGCAGAt                                                                                                                                                                                                  <  1:687065/281‑1 (MQ=255)`  
                                                                                                          `ataagagacagcAACAGAAGAAACCTGAACGCCAGCACAGGAGGCCGCACGCACCCGAGCTGCTAACAAAGCCCGAAAGGAAGCTGAGTTGGCTGCTGCCACCGCTGAGCAATCACTAGCATAACCCCTTGGGGCCTCTAAACGGGTCTTGAGGGGTTTTTTGCTGAAAGGAGAAACTATATGCGCTCATGCTATATGAACGTTGAGACTGCCGCTGAGTTATCAGCTGTGAACGACATTCTGGCGTCTATCGGTGAACCGCCGGTATCAACGCTGGAAGGTGACGCTAACGCAGATGCAg                                                                                                                                                                                              <  1:80732/290‑1 (MQ=255)`  
                                                                                                            `gtgtTACTTCAACAGAAGAAACCTTAACGCCAGCACAGGAGGCCGCACGCACCCTCGCTGCTAACAAAGCCCGAAAGGAAGCTGAGTTGGCTGCTGCCACCGCTGAGCAATCACTAGCATAACCCCTTGGGGCCTCTAAACGGGTCTTGAGGGGTTTTTTGCTGAAAGGAGGAACTATATGCGCTCATGCGATATGAACGTTGAGACTGCCGCTGAGTTATCAGCTGTGAACGCCATTCTGGCGTCTATCGGTGAACCTCCGGTATCAACGCTGGACGGTTACGCTGACGCAGATTCAGCg                                                                                                                                                                                            >  1:765231/1‑301 (MQ=255)`  
                                                                                                            `gtgtTACTTCAACAGAAGAAACCTTAACGCCAGCACAGGAGGCCGCACGCACCCGCGCTGCTAACAAAGCCCGAAAGGAAGCTGAGTTGGCTGCTGCCACCGCTGAGCAATCACTAGCATAACCCCTTGGGGCCTCTAAACGGGTCTTGAGGGTTTTTTTGCTGAAAGGAGGAACTATATGCGCTCATGCGATATGAACGTTGAGACTGCCGCTGAGTTATCAGCTGTGAACGACATTCTGGCGTCTATCGGTGAACCTCCGGTATCAACGCTGGAAGGTTACGCTAACGCAGATGCAGCg                                                                                                                                                                                            >  1:52384/1‑301 (MQ=255)`  
                                                                                                            `gtgtTACTTCAACAGAAGAAACCTTAACGCCAGCACAGGAGGCCGCACGCACCCGCGCTGCTAACAAAGCCCGAAAGGAAGCTGAGTTGGCTGCTGCCACCGCTGAGCAATCACTAGCATAACCCCTTGGGGCCTCTAAACGGGTCTTGAGGGGTTTTTTGCTGAAAGGAGGAACTATATGCGCTCATGCGATATGAACGTTGAGACTGCCGCTGAGTTATCAGCTGTGAACGACATTCTGGCGTCTATCGGTGAACCTCCGGTATCAACGCTGGAAGGTGACGCTAACGCAGATGCAGCg                                                                                                                                                                                            <  1:615705/301‑1 (MQ=255)`  
                                                                                                              `cgtgggctcggagatgtgtataagagacaggCACAGGAGGCCGCACGCACCCGCGCTGCTAACAAAGCCCGAAAGGAAGCTGAGTTGGCTGCTGCCACCGCTGAGCAATCACTAGCATAACCCCTTAGGGCCTCTAAACGGGTCTTGAGGGGTTTTTTGCTGAAAGGAGGAACTATATGCGCTCATGCGATATGAACGTTGAGACTGCCGCTGAGTTATCAGCTGTGAACGACATTCTGGCGTCTATCGGTGAACCTCCGGTATCAACGCTGGAAGGTGACGCTAACGCAGATGCAGCGaa                                                                                                                                                                                          <  1:39537/271‑1 (MQ=255)`  
                                                                                                               `gatgtgtataagagacagccTTAACGCCAGCACAGGAGGCCGCACACACCCGCGCTGCTAACAAAGCCCGAAAGGAAGCTGAGTTGGCTGCTGCCACCGCTGAGCAATCACTAGCATAACCCCTTGGGGCCTCTAAACGGGTCTTGAGGGGTTTTTTGCTGAAAGGAGGAACTATATGCGCTCATGCGATATGAACGTTGAGACTGCAGCTGAGTTATCAGCTGTGAACGACATTCTGGCGTCTATCGGTGAACCTCCGGTATCAACGCTGGAAGGTGACGCTAACGCAGATGCAGCGAAc                                                                                                                                                                                         <  1:155278/283‑1 (MQ=255)`  
                                                                                                                     `cAACAGAAGAAACCTTAACGCCAGCACAGGAGGCCGCACGCACCCGCGCTGCTAACAAAGCCCGAAAGGAAGCTGAGTTGGCTGCTGCCACCGCTGAGCAATCACTAGCATAACCCCTTGGGGCCTCTAAACGGGTCTTGAGGGGTTTTTTGCTGAAAGGAGGAACTATATGCGCTCATGCGATATGAACGTTGAGACTGCCGCTGAGTTATCAGCTGTGAACGACATTCTGGCGTCTATCGGTGAACCTCCGGTATCAACGCTGGAAGGTGACGCTAACGCAGATGCAGCGAACGCTCgg                                                                                                                                                                                   >  1:652100/1‑301 (MQ=255)`  
                                                                                                                                      `aCGCCAGCACAGGAGGCCGCACGCACCCGCGCTGCTAACAAAGCCCGAAAGGAAGCTGAGTTGGCTGCTGCCACCGCTGAGCAATCACTAGCATAACCCCTTGGGGCCTCTAAACGGGTCTTGAGGGGTTTTTTGCTGAAAGGAGGAACTATATGCGCTCATGCGATATGAACGTTGAGACTGCCGCTGAGTTATCAGCTGTGAACGACATTCTGGCGTCTATCGGTGAACCTCCGGTATCAACGCTGGAAGGTGACGCTAACGCAGATGCAGCGAACGCTCGGCGTATTCTCAACAAGAt                                                                                                                                                                  <  1:437206/301‑1 (MQ=255)`  
                                                                                                                                       `cGCCAGCACAGGAGGCCGCACGCACCCGCGCTGCTAACAAAGCCCGAAAGGAAGCTGAGTTGGCTGCTGCCACCGCTGAGCAATCACTAGCATAACCCCTTGGGGCCTCTAAACGGGTCCTGAGGGGTTTTTTGCTGAAAGGAGGAACTATATGCGCTCATGCGATATGAACGTTGAGACTGCCGCTGAGTTATCAGCTGTGAACGACATTCTGGCGTCTATCTGTGAACCTCCGGTATCAACGCTGGCAGGTGACGCTAACGCAGATGCAGCGAACGCTCGGCGTATTCTCCACAAGAtt                                                                                                                                                                 >  1:421353/1‑301 (MQ=255)`  
                                                                                                                                            `gCACAGGAGGCCGCACGCACCCGCGCTGCTAACAAAGCCCGAAAGGAAGCTGAGTTGGCTGCTGCCACCGCTGAGCAATCACTAGCATAACCCCTTGGGGCCTCTAAACGGGTCTTGAGGGGTTTTTTGCTGAAAGGAGGAACTATATGCGCTCATGCGATATGAACGTTGAGACTGCCGCTGAGTTATCAGCTGTGAACGACATTCTGGCGTCTATCGGTGAACCTCCGGTATCAACGCTGGAAGGTGACGCTAACGCAGATGCAGCGAACGCTCGGCGTATTCTCAACAAGAtctgtct                                                                                                                                                            >  1:657355/1‑295 (MQ=255)`  
                                                                                                                                              `acaGGAGGCCGCATGCACCCGCGCTGCTAACAAAGCCCGAAAGGAAGCTCAGTTGGCTGCTGCCACCGCTGAGCAATCACTAGCATAACCCCTTGGGGCCTCTAAACGGGTCTTGAGGGGTTTTTTGCTGAAAGGAGAAACTATATGCGCTCATGCGATATGAACGTTGAGACTGCCGCTGAGTTATCAGCTGTGAACGACATTCTGGCGTCTATCGGTGAACCTCCGGTATCAACGCTGGAAGGTGACGCTAACGCAGATGCAGCGAACGCTCGGCGTATTCTCAACAAGATTAACCGAc                                                                                                                                                          >  1:333004/1‑301 (MQ=255)`  
                                                                                                                                               `caGGAGGCCGCACGCACCCGCGCTGCTAACAAAGCCCGAAAGGAAGCTGAGTTGGCTGCTGCCACCGCTGAGCAATAACTAGCATAACCCCTTTGGGCCTCTAAACTGGTCTTGAGGGGTTTTTTGCTGAAAGGAGGAACTATATGCGCTCATGCGATATGAACGTTGAGACTGCCGCTGAGTTATCAGCTGTGAACGACATTCTGGCGTCTATTGGTGAACCTCCGGTATCAACGCTGGAAGGTGACGCTAACGCAGATGCAGCGAACGCTCGGCGTATTCTgaacctgtctcttataca                                                                                                                                                         >  1:754428/1‑287 (MQ=255)`  
                                                                                                                                                        `gcacgcacCAGCGCTGCTAACAAAGCCCGAAAGGAAGCTGAGTTGGCTGCTGCCACCGCTGAGCAATAACTAGCATAACCCCTTGGGGCCTCTAAACGGGTCTTGAGGGGTTTTTTGCTGAAAGGAGGAACTATATGCACTCATGCGATATGAACGTTGAGACTGCCGCTGAGTTATCAGCTGTGAACGACATTCTGGCGTCTATCGGTGAACCTCCGGTATCAACGCTGGAAGGTGACGCTAACGCAGATGCAGCGAACGCTCGGCGTATTCTCAACAAGATTAACCGACAGATTCAAtc                                                                                                                                                <  1:135132/301‑1 (MQ=255)`  
                                                                                                                                                         `cgtcagatgtgtataagagacaggCCCGAAAGGAAGCTGAGTTGGCTGCTGCCACCGCTTAGCAATCACTAGCATAACCCCTTGGGGCCTCTAAACGGGTCTTGAGGGGTTTTTTGCTGAAAGGAGGAACTATATGCGCTCATGCGATATGAACGTTGAGACTGCCGCTGAGTTATCAGCTGTGAACGACATTCTGGCGTCTATCGGTGAACCTCCGGTATCAACGCTGGAAGGTGACGCTAACGCAGATGCAGCGAACGCTCGGCGTATTCTCAACAAGATTAACCGACAGCTTCAAtct                                                                                                                                               >  1:339872/24‑301 (MQ=255)`  
                                                                                                                                                         `cactcacCCGCGCTGCTAACAAAGCCCGAAAGGAAGCTGAGTTGGCTGCTGCCACCGCTGAGCAATCACTAGCATAACCCCTTGGGGCCTCTAAACGGGTCTTGAGGGGTTTTTTGCTGAAAGGAGGAACTATATGCGCTCATGCGATATGAACGTTGATACTGCCGCTGAGTTATCAGCTGTGAACGACATTCTGGCGTCTATCGGTGAACCTCCGGTATCAACGCTGGAAGGTGACGCTAACGCAGATGCAGCGAACGCTCGGCGTATTCTCAACAAGATTAACCGACAGATTCAAtct                                                                                                                                               <  1:545628/301‑1 (MQ=255)`  
                                                                                                                                                           `cgcacCCGCGCTGCTAACAAAGCCCGAAAGGAAGCTGAGTTGGCTGCTGCCACCGCTGAGCAATCACTAGCATAACCCCTTGGGGCCTCTAAACGGGTCTTGAGGGGTTTTTTGCTGAAAGGAGGAACTATATGAGCTCATGCGATATGAACGTTGAGACTGCCGCTGAGTTATCAGCTGTGAACGACATTCTGGCGTCTATCGGTGAACCTCCGGTATCAACGCTGGAAGGTGACGCTAACGCAGATGCAGCGAACGCTCGGCGTATTCTCAACAAGATTAACCGACAGATTCAAtctcc                                                                                                                                             >  1:375910/1‑300 (MQ=255)`  
                                                                                                                                                                 `cgcgCTGCTAACAAAGCCCGAAAGGAAGCTGAGTTGGCTGCTGCCACCGCTGAGCAATAACTAGCATATCCCCTTGGGGCCTCTAAACGGGTCTTGAGGGGTTTTTTGCTGAAAGGAGGAACTATATGCGCTAATGCAATATGAACGTTGAGACTGCAGCTGAGTTATCAGCTGTGAACGACATTCTGGCGTCTATCGGTGATCCTCCGGCATCAACGCTAGAAGGTGACGCTAACGCAGATGCAGCGAACGCTCGGCGTATTCTCAACAAGATTAACCGACAGATTCAATCTCGtggatg                                                                                                                                       <  1:47132/301‑1 (MQ=255)`  
                                                                                                                                                                                  `cggagatgtgtataagagacaggctgcCACCGCTGAGCAATCACTAGCATAACCCCTTGGGGCCTCTAAACGGGTCTTGAGGGGTTTTTTGCTGAAATGAGGAACTATATGCGCTCATGCGATATGAACGTTGAGACTGCCGCTGAGTTATCAGCTGTGAACGACATTCTGGCGTCTATCGGTGAACCTCCGGTATCAACGCTGGAAGGTGACGCTAACGCAGATGCAGCGAACGCTCGGCGTATTCTCAACAAGATTAACCGACAGATTCAATCTCGTGGATGGACGTTCAACATTGAgg                                                                                                                      <  1:653232/279‑1 (MQ=255)`  
                                                                                                                                                                                     `aaaGGAAGCTGAGTTGGCTGCTGCCACCGCTGAGCAATCACTAGCATAACCCCTTTGGGCCTCTAAACGGGTCTTGAGGGGTTTTTTGCTGAAAGGAGGAACTATATGCGCTCATGCGATATGAACGTTGAGACTGCCGCTGAGTTATCAGCTGTGAACGACATTCTGGCGTCTATCGGTGAACCTCCGGTATCAACGCTGGAAGGTGACGCTAACGCAGATGCAGCGAACGCTCGGCGTATTCTCAACAAGATTAACCGACAGATTCAAtcctgtctcttatacacatctccgagcccac                                                                                                                   >  1:670597/1‑272 (MQ=255)`  
                                                                                                                                                                                     `aaaGGAAGCTGAGTTGGCTGATGCCACCGCTGAGCAATCACTAGCATAACCCCTTGGGGCCTCTAAACGGGTCTTGAGGGGTTTTTTGCTGAAAGGAGGAACTATATGCGCTCATGCGATATGAACGGTGAGACTGCCGCTGAGTTATCAGCTGTGAACGACATTCTGGCGTCTATCGGTGAACCTCCGGTATCAACGCTGGAAGGTGACGCTAACGCAGATGCAGCGAACGCTCGGCGTATTCTCAACAAGATTAACCGACAGATTCAATCTCGTAGATGGACGTTCAACCTTGAggcgg                                                                                                                   >  1:103493/1‑298 (MQ=255)`  
                                                                                                                                                                                             `cTGAGTTGGCTGCTGCCACCGCTGAGCAATCACTAGCATAACCCCTTGGGGCCTCTAAACGGGTCTTGAGGGGTTTTTTGCTGAAAGTAGGAACTATATGCGCTTCTGCGATATGAACGTTGAGACTGCCGCTGAGTTATTAGCTGTGAACGACATTCTGGCGTCTATCGGTGAACCTCCGGTATCAACGCTGGTAGGTGACGCTAACGCATATGCAGCGAACGCTCGGCGTATTCCCACCAAGATTAACTGACAGATTCAATCTCGTGGATGGACGTTCAACATTGAGGAAGGCATAACg                                                                                                           >  1:51975/1‑301 (MQ=255)`  
                                                                                                                                                                                                 `gTTGGCTGCTGCCACCGCTGAGCAATCACTAGCATAACCCCTTGGGGCCTCTAAACGGGTCTTGAGGGGTTTTTTGCTGAAAGGAGGAACTATATGCGCTCATGCGATATGAACGTTGAGACTGCCGCTGAGTTATCAGCTGTGAACGACATTCTGGCGTCTATCGGTGAACCTCCGGTATCAACGCTGGAAGGTGACGCTAACGCAGATGCAGCGAACGCTCGGCGTATTCTCAACAAGATTAACCGACAGATTCAATCTCGTTGATGGACGTTCAACATTGAGGAAGGCATAACGctac                                                                                                       >  1:639285/1‑301 (MQ=255)`  
                                                                                                                                                                                                   `tGGCTGCTGCCCCCGCTGAGCAATCACTAGCATAACCCCCTGGGGCCTCTAAACGGGTCTTGAGTGGTTTTTTGCTGAAAGGAGGAACTATATGCGCTCATGCGATATGAACGTTGATACTGCCGCTGAGTTATCAGCTGTGAACGACATTCTGGCGTCTATCGGTGAACCTCCGGTATCAACGCTGGAAGGTGACGCTAACGTAGATGCAGCGAACGCTCGGCGTATTCTCAACAAGATTAACCGACAGATTCAATCTCGTGGATGGACGTTCAACATTGAGGAAGGCATAACGctacta                                                                                                     <  1:809908/301‑1 (MQ=255)`  
                                                                                                                                                                                                          `aggtgtataagagacagcaCTAGCATAACCCCTTGGGGCCTCTAAACGGGTCTTGAGGGGTTTTTTGCTGAAAGGAGGAACTATATGCGCTCATGCGATATGAACGTTGAGACTGCCGCTGAGTTATCAGCTGTGAACGACATTCTGGCGTCTATCGGTGAACCTCCGGTATCAACGCTGGAAGGTGACGCTAACGCAGATGCAGCGAACGCTCGGCGTATTCTCAACAAGATTAACCGACAGATTCAATCTCGTGGATGGACGTTCAACATTGAGGAAGGCATAACGCTACTACCTGATg                                                                                              <  1:112091/283‑1 (MQ=255)`  
                                                                                                                                                                                                           `ggagatgtgtataagagacaggCATAACCCCTTGGGGCCTCTAAACGGGTCTTGAGGGGTTTTTTGCTGAAAGTAGGAACTATATGCGCTCATGCGATATGAACGTTGATACTGCCGCTGAGTTATCAGCTGTGAACGACATTCTGGCGTCTATCGGTGAACCTCCTGTATCAACGCTGGAAGGTGACGCTAACGCAGATGCAGCGAACGCTCGGCGTATTCTCAACAAGATTAACCGACAGATTCAATCTCGTGGATGGACGTTCAACATTGAGGAAGGCATAACGCTACTACCTGATGt                                                                                             <  1:209338/280‑1 (MQ=255)`  
                                                                                                                                                                                                               `cctcTGAGCAATCACTAGCATAACCCCTTGGGGCCTCTAAACGGGTCTTGAGGGGTTTTTTGCTGAAAGGAGGAACTATATGCGCTCATGCGATATGAACGTTGAGACTGCCGCTGAGTTATCAGCTGTGAACGACATTCTGGCGTCTATCGGTGAACCTCCGGTATCAACGCTGTAAGGTGACGCTAACGCAGATGCAGCGAACGCTCGGCGTATTCTCAACAAGATTAACCGACAGATTCAATCTCGTGGATGGACGTTCAACATTTAGGAAGGCATAACGCTACTACCTGATGTTTaa                                                                                         >  1:718162/4‑300 (MQ=255)`  
                                                                                                                                                                                                               `ccGCTGAGCAATCACTAGCATAACCCCTTGGGGCCTCTAAACGGGTCTTGAGGGGTTTTTTGCTGAAAGGAGGAACTATATGCGCTCATGCGATATGAACGTTGAGACTGCCGCTGAGTTATCAGCTGTGAACGACATTCTGGCGTCTATCGGTGAAACTCCGGTATCAACGCTGGAAGGTGACGCTAACGCAGATGCAGCGAACGCTCGGCGTATTCTCAACAAGATTAACCGACAGATTCAATCTCGTGGATGGACGTTCAACATTGAGGAAGGCATAACGCTACTACCTGATGTTTAc                                                                                         >  1:624969/1‑301 (MQ=255)`  
                                                                                                                                                                                                                    `gAGCAATCACTAGCATAACCCCTTGGGGCCTCTAAACGGGTCTTGAGGGGTTTTTTGCTGAAAGGAGGAACTATATGCGCTCATGCGATATGAACGTTGAGACTGCCGCTGAGTTATCAGCTGTGAACGACATTCTGGCGTCTATCGGTGAACCTCCGGTATCAACGCTGGCAGGTGAAGCTAACGCAGATGCAGCGAACGCTCGGCGTATTCTCAACAAGATTAACCGACAGATTCAATCTCGTGGATGGACGTTCAACATTGAGGAAGGCATAACGCTACTACCTGATGTTTACTACaa                                                                                    >  1:142200/1‑301 (MQ=255)`  
                                                                                                                                                                                                                      `gCAATCACTAGCATAACCCCTTGGGGCCTCTAAACGGGTCTTGAGGGGTTTTTTGCTGAAAGGAGGAACTATATGCGCTCATGCGATATGAACGTTGAGACTGCCGCTGAGTTATCAGCTGTGAACGACATTCTGGCGTCTATCGGTGAACCTCCGGTATCAACGCTGGAAGGTGACGCTAACGCAGATGCAGCGAAAGCTCGGCGTATTCTCAACAAGATTAACCGACAGATTCAATCTCGTGGATGGACGTTCAACATTGAGGAAGGCATAACGCTACTACCTGATGTTTACTCCAAcc                                                                                  <  1:641304/301‑1 (MQ=255)`  
                                                                                                                                                                                                                      `atgtgtataagagacagcccTTGGGGCCTCTAAACGGGTCTTGAGTGGTTTTTTGCTGAAAGGAGGAACTATATGCGCTCATGCGATATGAACGTTGAGACTGCCGCTGAGATATCAGCTGTGAACGACATTCTGGCGTCTATCGGTGAACCTCCGGTATCAACGCTGGAAGGTGACGCTAACGCAGATGCAGCGAACGCTCGGCGTATTCTCAACAAGATTAACCTACAGATTCAATCTCGTGGATGGACGTTCAACATTGAGGAAGGCATAACGCTACTACCTGATGTTTACTCCAAcc                                                                                  <  1:181880/284‑1 (MQ=255)`  
                                                                                                                                                                                                                         `atcaCTAGCATAACCCCTTGGGGCCTCTAAACGGGTCTTGCGGGGTTTTTTGCTGAAAGGAGGAACTATATGCGCTCATGCGATATGAACGTTGAGACTGCCGCTGAGTTATCAGCTGTGAACGACATTCTGGCGTCTATCGGTGAACCTCCGGTATCAACGCTGGAAGGTGACGCTAACGCAGATGCAGCGAACGCTCGTCGTATTCTCAACAAGATTAACCGACAGATTCAATCTCGTGGATGGACGTTCAACATTGAGGAAGGCATAACGcctgtctcttataaacatctccgagccc                                                                               >  1:255876/4‑274 (MQ=255)`  
                                                                                                                                                                                                                          `tAACTAGCATAACCCCTTGGGGCCTCTAAACGGGTCTTGAGGGGTTTTTTGCTGAAAGGAGGAACTATATGCGCTCATGCGATATGAACGTTGAGACTGCCGCTGAGTTATCAGCTGTGAACGCCATTCTGGCGTCTATCGGTGAACCTCCGGTATCAACGCTGGAAGGTGACGCTAACGCAGATGCAGCGAACGCTCGGCGTATTCTCAACAAGATTAACCGACAGATTCAATCTAGTGGATGGACGTTCAACATTGAGGAAGGCATAACGCTACTACCTGATGTTTACTCCAACCTGAt                                                                              >  1:583329/1‑301 (MQ=255)`  
                                                                                                                                                                                                                          `tAACTAGCATAACCCCTTGGGGCCTCTAAACGGGTCTTGAGGGGTTTTTTGCTGAAAGGAGGAACTATATGCGCTCATGCGATATGAACGTTGAGACTGCCGCTGAGTTATCAGCTGTGAACGACATTCTGGCGTCTATCGGTGAACCTCCGGTATCAACGCTGGAAGGTGACGCTAACGCAGATGCAGCGAACGCTCGGCGTATTCTAAACAAGATTAACCGACAGATTCAATCTAGTGGATGGACGTTCAACATTGAGGAAGGCATAACGcctgtctcttatacacatctccgagcccc                                                                              >  1:505809/1‑273 (MQ=255)`  
                                                                                                                                                                                                                           `caCTAGCATAACCCCTTGGGGCCTCTAAACGGGTCTTGAGGGGTTTTTTGCTGCAAGGAGGAACTATATGCGCTCATGCGATATGAACGTTGAGACTGCCGCTGAGTTATCAGCTGTGAACGTCATTCTGGCGTCTATCGGTGAACCTCCGGTATCAACGCTGGAAAGTGACGCCAACGCAGATGCCGCGAACGCTCGGCGTATTCTCAACAAGATTAACCGACAGATTCAATCTCGTGGATGGACGTTCAACATTGAGGCAGGCATAACGCTACTCCCTGATGTTTACTCCAACCTGAtt                                                                             >  1:5720/2‑301 (MQ=255)`  
                                                                                                                                                                                                                                `gCATAACCCCTTGGGGCCTCTAAACGGGTCTTGAGGGGTTTTTTGCTGAAAGGAGGAACTATATGCGCTCATGCGATATGAACGTTGAGACTGCCTCTGAGTTATCAGCTGTGAACGACATTCTGGCGTCTATCGGTGAACCTCCGGTATCAACGCTGGAAGGTTACGCTAACGCAGATGCAGCGAACGCTCGGCGTATTCTCAACAAGATTAACCGACAGATTCAATCTCGTGGATGGACGTTCAACATTGAGGAAGGCATAACGCTACTACCTGATGTTTACTCCAACCTGATTGtatt                                                                        >  1:735726/1‑300 (MQ=255)`  
                                                                                                                                                                                                                                 `tgggctcggagatgtgtataagagacagcTTGAGTGGTTTTTTGCTGAAAGGAGGATATATATGCGCTCATGCGATATGAACGTTGAGACTGCCGCTGAGTTATCAGCTGTGAACGACATTCTGGCGTCTATCGGTGAACCTCCGGTATCAACGCTGGAAGGTGACGCTAACGCAGATGCAGCGAACGCTCGGCGTATTCTCAACAAGATTAACCGACAGATTCAATCTCGTGGATGGACGTTCAACATTGAGGAAGGCATAACGCTACTACCTGATGTTTACTCCAACCTGATTGTATAc                                                                       <  1:192347/273‑1 (MQ=255)`  
                                                                                                                                                                                                                                 `gagatgtgtataagagacagaaaCGGGTCTTGAGGGGTTTTTTGCTGAAAGGAGGAACTATATGCGCTCATGCGATATGAACGTTGAGACTGCCGCTGAGTTATCAGCTGTGAACGACATTCTGGCGTCTATCGGTGAACCTCCGGTATCAACGCTGGAAGGTGACGCTAACGCAGATGCAGCGAACGCTCGGCGTATTCTCAACAAGATTAACCGACAGATTCAATCTCGTGGATGGACGTTCAACATTGAGGAAGGCATAACGCTACTACCTGATGTTTACTCCAACCTGATTGTATAc                                                                       <  1:88851/281‑1 (MQ=255)`  
                                                                                                                                                                                                                                 `cATAACCCCTTGGGGCCTCTAAACGGGTCTTGAGGGGTTTTTTGCTGAAAGGAGGAACTATATGCGCTCATGCGATATGAACGTTGAGACTGCCGCTGAGTTATCAGCTGTGAACGACATTCTGGCGTCTATCGGTGAACCTCCGGTATCAACGCTGGAAGGTGACGCTAACGCAGATGCAGCTAACGCTCGGCGTATTCTCAACAAGATTAACCGACAGATTCAATCTCGTGGATGGACGTTCACCCTTGCGGACGTCATACCGCTACTCCCTGATGTTTACTCCAACCTGATTGTATAc                                                                       <  1:287315/301‑1 (MQ=255)`  
                                                                                                                                                                                                                                 `cATAACCCCTTGGGGCCTCTAAACGGGTCTTGAGGGGTTTTTTGCTGAAAGGAGGAACTATATGCGCTCATGCGATATGAACGTTGAGACTGCCGCTGAGTTATCAGCTGTGAACGACATTCTGGCGTCTATCGGTGAACCTCCGGTATCAACGCTGGAAGGTGACGCTAACGCAGATGCAGCGAACGCTCGGCGTATTCTCAACAAGATTAACCGACAGATTCAATCTCGTGGATGGACGTTCAACATTGAGGAAGGCATAACGCTACTACCTGATGTTTACTCCAACCTGATTGTATAc                                                                       <  1:281842/301‑1 (MQ=255)`  
                                                                                                                                                                                                                                 `cATAACCCCTTGGGGCCTCTAAACGGGTCTTGAGGGGTTTTTTGCTGAAAGGAGGAACTATATGCGCTCATGCGATATGAACGTTGAGACTGCCGCTGAGTTATCAGCTGTGAACGACATTCTGGCGTCTATCGGTGAACCTCCGGTATCAACGCTGGAAGGTGACGCTAACGCAGATGCAGCGAACGCTCGGCGTATTCTCAACAAGAATAACCGACAGATTCAATCTCGTGGATGGACGTTCAACATTGAGGAATGCATAACGCTACTACCTGATGTTTACTCCAACCTGATTGTATAc                                                                       <  1:504174/301‑1 (MQ=255)`  
                                                                                                                                                                                                                                   `tcggagatgtgtataagagacagggTCTTGAGGGGTTTTTTGCTGAAAGGAGGAACTATATGCGCTCATGCGATATGAACGTTGAGACTGCCGCTGAGTTATCAGCTGTGAACGACATTCTGGCGTCTATCGGTGAACCTCCGGTATCAACGCTGGAAGGTGACGCTAACGCAGATGCAGCGAACGCTCGGCGTATTCTCAACAAGATTAACCGACAGATTCAATCTCGTGAATGGACGTTCAACATTGAGGAAGGCATAACGCTACTACCTGATGTTTACTCCAACCTGATTGTATACAg                                                                     <  1:767593/279‑1 (MQ=255)`  
                                                                                                                                                                                                                                   `aaaCCCCTTGGGGCCTCTAAACGGGTCTTGAGGGGTTTTTTGCTGAAAGGAGGAACTATATGCGCTCATGCGATATGAACGTTGAGACTGCCGCTGAGTTATCAGCTGTGAACGACATTCTGGCGTCTATCGGTGAACCTCCGGTATCAACGCTGGAAGGTGACGCTAACGCAGATGCAGCGAACGCTCGGCGTATTCTCAACAAGATTAACCGACAGATTCAATCTCGTGGATGGACGTTCAACATTGAGGAAGGCATAACGCTACTACCTGATGTTTACTCCAACCTGATTGTATACAg                                                                     <  1:680742/300‑1 (MQ=255)`  
                                                                                                                                                                                                                                     `aatgtgtataagagacagaCGGGTCTTGAGGTGTTTTTTGCTGAAAGGAGGAACTATATGCGTTCATGCGATATGAACGTTGAGACTGCCGCTGAGTTATCAGCTGTGAACGACATTCTGGCGTCTATCGGTGAACCTCCGGTATCAACGCTGGAAGGTGACGCTAACGCAGATGCAGCGAACGCTCGGCGTATTCTCAACAAGATTAACCGACAGATTCAATCTCGTGGATGGACGTTCAACATTGAGGAAGGCATAACGCTACTACCTGATGTTTACTCCAACCTGATTGTATACAGTg                                                                   <  1:291830/283‑1 (MQ=255)`  
                                                                                                                                                                                                                                     `aCCCCTTGGGGCCTCTAAATGGGTCTTGAGGGGTTTTTTGCTGAAAGGAGGAACTATATGCGCTCATGCGATATGAACGTTGAGACTGCCGCTGAGTTATCAGCTGTGAACGACATTCTGGCGTCTATCAGTGAACCTCCGGTATCAACGCTGGAAGGTGACGCTAACGCAGATGCAGCGAACGCTCGGCGTATTCTCAACAAGATTAACCGACAGATTCAATCTCGTGGATGGACGTTCAACATTGAGGAAGGCATAACGCTACTACCTGATGTTTACTCCAACCTGATTGTATACAGTg                                                                   >  1:346961/1‑301 (MQ=255)`  
                                                                                                                                                                                                                                     `aCCCCTTGGGGCCTCTAAACGGGTCTTGAGGGGTTTTTTGCTGAAAGGAGGAACTATATGCGCTCATGCGATATGAACGTTGAGACTGCCGCTGAGTTATCAGCTGTGAACGACATTCTGGCGTCTATCGGTGAACCTCCGGTATCAACGCTGGAAGGTGACGCTAACGCAGATGCAGCGAACGCTCGGCGTATTCTCAACAAGATTAACCGACAGATTCAATCTCGTGGATGGACGTTCAACATTGAGTAAGGCATAACGCTAATACCTGATGTTTACTTCAAccctgtctcttatacaa                                                                   >  1:695912/1‑286 (MQ=255)`  
                                                                                                                                                                                                                                     `aCCCCTTGGGGCCTCTAAACGGGTCTTGAGGGGTTTTTTGCTGAAAGGAGGAACTATATGCGCTCATGCGATATGAACGTTGAGACTGCCGCTGAGTTATCAGCTGTGAACGACATTCTGGCGTCTATCGGTGAACCTCCGGTATCAACGCTGGAAGGTGACGCTAACGCAGATGCAGCGAACGCTCGGCGTATTCTCAACAAGATTAACCGACAGATTCAATCTCGTGGATGGACGTTCAACATTGAGGAAGGCATAACGCTACTACCTGATGTTTACTCCAACCTGATTGTATACAGtt                                                                   >  1:277857/1‑300 (MQ=255)`  
                                                                                                                                                                                                                                       `cccTTGGTGCCTCTAAACGGGTCTTGAGGGGTTTTTTGCTGAAAGGAGGAACTATATGCGCTCATGCGATATGAACGTTGAGACTGCCGCTGAGTTATCAGCTGTGAACGACATTCTGGCGTCTATCGGTGAACCTCCGGTATCAACGCTGGAAGGTGACGCTAACGCAGATGCAGCGAACGCTCGGCGTATTCTCAACAAGATTAACCGACAGATTCAATCTCGTGGATGGACGTTCAACATTGAGGAAGGCATAACGCTACTACCTGATGTTTACTcctgtctcttatacacatctccg                                                                 >  1:496491/1‑280 (MQ=255)`  
                                                                                                                                                                                                                                       `cccTTGGGGCCTCTAAACGGGTCTTGAGGGGTTTTTTGCTGAAAGGAGGAACTATATGCTCTCATGCGATATGAACGTTGAGACTGCCGCTGAGTTATCAGCTGTGAACGACATTCTGGCGTCTATCGGTGAACCTCCGGTATCAACGCTGGAAGGTGACGCTAACGCAGATGCAGCGAACGCTCGGCGTATTCTCAACAAGATTAACCGACAGATTCAATCTCGTGGATGGACGTTCAACATTGAGGAAGGCATAACGCTACTACCTGTTgctgtctcttatacacatctccgagcccac                                                                 >  1:386196/1‑272 (MQ=255)`  
                                                                                                                                                                                                                                       `cccTTGGGGCCTCTAAACGGGTCTTGAGGGGTTTTTTGCTGAAAGGAGGAACTATATGCGCTCATGCGATATGAACGTTGAGACTGCCGCTGAGTTATCAGCTGTGAACGACATTCTGGCGTCTATCGGTGAACCTCCGGTATCAACGCTGGAAGGTGACGCTAACGCAGATGCAGCGAACGCTCGGCGTATTCTCAACAAGATTAACCGACAGATTCAATCTCGTGGATTGACGTTCAACATTGAGGAAGGCATAACGCTACTACCTGATGTTTACTccctgtctcttatacacatctcc                                                                 >  1:92929/1‑280 (MQ=255)`  
                                                                                                                                                                                                                                       `cccTTGGGGCCTCTAAACGGGTCTTGAGGGGTTTTTTGCTGAAAGGAGGAACTATATGCGCTCATGCGATATGAACGTTGAGACTGCCGCTGAGTTATCAGCTGTGAACGACATTCTGGCGTCTATCGGTGAACCTCCGGTATCAACGCTGGAAGGTGACGCTAACGCAGATGCAGCGAACGCTCGGCGTATTCTCAACAAGATTAACCGACAGATTCAATCTCGTGGATGGACGTTCAACATTGAGTAAGGCATAACGCTACTACCTGATGTTTACTCCAACCTGATTGTATAcctgtct                                                                 >  1:531827/1‑295 (MQ=255)`  
                                                                                                                                                                                                                                        `ccTTGGGGCCTCTAAACGGGTCTTGAGGGGTTTTTTGCTGAAAGGAGGAACTATATGCGCTCATGCGATATGAACGTTGAGACTGCCGCTGAGTTATCAGCTGTGAACGACATTCTGGCGTCTATCGGTGAACCTCCGGTATCAACGCTGGAAGGTGACGCTAACGCAGATGCAGCGAACGCTCGGCGTATTCTCAACAAGATTAACCGACAGATTCAATCTCGTGGATGGACGTTCAACATTGAGGAAGGCATAACGCTACTACCTGATGTTTACTCCAACCTGATTGTATACAGTgacc                                                                >  1:597941/1‑300 (MQ=255)`  
                                                                                                                                                                                                                                               `gCCTCTAAACGGGTCTTGAGGGGTTTTTTGCTGAAAGGAGGAACTATATGCGCTCATGCGATATGAACGTTGAGACTGCCGCTGAGTTATCAGCTGTGAACGACATTCTGGCGTCTATCGGTGAACCTCTGGTATCAACGCTGGAAGGTGACGCTAACGCAGATGCAGCGAACGCTCGGCGTATTCTCAACAAGATTAACCGACAGATTCAATCTCGTGGATGGACGTTCAACATTGAGGAAGGAATAACGCTACTACCTGATGTTTACTCCAACCTGATTGTATACAGTGACGACTAttt                                                         <  1:81705/301‑1 (MQ=255)`  
                                                                                                                                                                                                                                                `ccTCTAAACGGGGCTTGAGGGGTTTGTTGCCGAAAGGAGGAACTATATGCGATCATGCGATATGAACGTTGAGACTGCCGCTGAGTTATCAGCTGTGAACGACATTCTGGCGTCTATCGGTGAACCTCCGGTATCAACGCTGGAAGGTGACGCTAACGCAGATGCAGCGAACGCTCGGAGTATTCTCAACAAGATTAACCGACAGATTCAATCTCGTGGATGGACGTTCAACATTGAGGAAGGCATAACGCTACTACCTGATGTTTACTCCAACCTGATTGTATACAGTGACGACTATTTa                                                        <  1:3517/301‑1 (MQ=255)`  
                                                                                                                                                                                                                                                 `ctctAAACTGGTCTTGAGGGGTTTTTTGCTGAAAGGAGGAACTATATGCGCTCATGCGATATGAACGTTGAGACTGCCGCTGAGTTATCAGCTGTGAACGACATTATGGCGTCTATCGGTGAACCTCCGGTATCAACGCTGGAAGGTGACGCTAACGCAGATGCAGCGAACGCTCGGCGTATTCTCAACAAGATTAACCGACAGATTCAATCTAGTGGATGGACGTTCAACATTGAGGAAGGCATAACGCTACTACCTGATGTTTACTCCAACCTGATTGTATACatgtctcttatacaca                                                       >  1:477347/1‑286 (MQ=255)`  
                                                                                                                                                                                                                                                    `tAAACGGGTCTTGAGGGGTTTTTTGCTGAAAGGAGGAACTATATGCGCTCATGCGATATGAACGTTGAGACTGCCGCGGAGTTATCAGCTGTGAACGACATTCTGGCGTCTATCGGTGAACCTCCGGTATCAACGCTGGAAGGTGACGCTAACGCAGATGCAGCGAACGCTCGGCGTATTCTCAACAAGATTAACCGACAGATTCAATATCGTGGATGGACGTTCAACATTGAGGAAGGCATAACGCTACTACCTGATGTTTACTCCAACCTGATTGTATACAGTGACGACTATTTATccc                                                    <  1:468216/301‑1 (MQ=255)`  
                                                                                                                                                                                                                                                    `ctcgttggctcggagatgtgtataagagacaggaggAACTATATGTGCTCATTCGATATGAACGTTGAGACTGCCGCTGAGTTATCAGCTGTGAACGACATTCTGGCGTCTATCGGTGAACCTCCGGTATCAACGCTGGAAGGTGACGCTAACGCAGATGCAGCGAACGCTCGGCGTATTCTCAACAAGATTAACCGACAGATTCAATCTCGTGGATGGACGTTCAACATTGAGGAAGGCATAACGCTACTACCTGATGTTTACTCCAACCTGATTGTATACAGTGACGACTATTTATccc                                                    <  1:750668/271‑1 (MQ=255)`  
                                                                                                                                                                                                                                                     `aaaCCGGTCTTGAGGGGTTTTTTGCTGAAAGGATGAACTATATGCGCTCATGCGATATGAACGTTGAGACTGCCGCTGAGTTATCAGCTGTGAACGACATTCTGGCGTCTATCGGTGAACCTCCGGTATCAACGCTGGAAGGTGACGCTAACGCAGATGCAGCGAACGCTCGGCGTATTCTCAACAAGATTAACCGACAGATTCAATCTCGTGGATGGACGTTCAACATTGAGGAAGGCATAACGCTACTACCTGATGTTTACTCCAACCTGATTGTATACAGTGACGACTATTTATCCCt                                                   >  1:725921/1‑301 (MQ=255)`  
                                                                                                                                                                                                                                                         `gggTCTTGATGGGTTTTTTGCTGAAAGGAGGAACTATATGCGCTCATGCGATATGAACGTTGAGACTGCCGCTGAGTTATCAGCTGTGAACGACATTCTGGCGTCTATCGGTGAACCTCCGGTATCAACGCTGGAAGGTGACGCTAACGCAGATGCAGCGAACGCTCGGCGTATTCTCAACAAGATTAACCGACAGATTCAATCTCGTGGATGGACGTTCAACATTGAGGAAGGCATAACGCTACTACCTGATGTTTACTCCAACCTGATTGTATAcctgtctcttatacacatctccgag                                               >  1:312530/1‑277 (MQ=255)`  
                                                                                                                                                                                                                                                          `gtgggctcggagatgtgtataagagacaggaACTATATGCGCTCATGCGATATGAACGTTGAGACTGCCGCTGAGTTATCAGCTGTGAACGACATTCTGGCGTCGATCGGTGAACCTCCGGTATCAACGCTGGAAGGTGACGCTAACGCAGATGCAGCGAACGCTCGGCGTATTCTCAACAAGATTAACCGACAGATTCAATCTCGTGGATGGACGTTCAACATTGAGGAAGGCATAACGCTACTACCTGATGTTTACTCCAACCTGATTGTATACAGTGACGACTATTTATCCATAATGt                                              <  1:549487/274‑1 (MQ=255)`  
                                                                                                                                                                                                                                                          `ggTCTTGAGGGGTTTTTTGCTGAAAGGAGGAACTATATGCGCTCATGCGATATGAACGTTGAGACTGCCGCTGAGTTATCAGCTGTGAACGACATTCTGGAGTCTATCGGTGAACCTCCGGTATCAACGCTGGAAGGTGACGCTAACGCAGATGCAGCGAACGCTCGGCGTATTCTCAACAAGATTAACCGACAGATTCAATCTAGTGGATGGACGTTCAACATTGAGGAAGGCATAACGCTACTACCTGATGTTTACTCCAACCTGATTGTATAcctgtctcttatacacatctccgagc                                              >  1:741459/1‑276 (MQ=255)`  
                                                                                                                                                                                                                                                          `ggTCTTGAGGGGTTTTTTGCTGAAAGGAGGAACTATATGCGCTAATGCGATATGAACGTTGAGACTGCCGCTGAGTTATCAGCTGTGAACGACATTCTGGCGTCTATCGGGGAACCTCCGATATCAACGCTGGAAGGTGACGCTAACGCAGATGCAGCGAACGCTCGGCGTATTCTCAACAAGATTAACCGACAGATTCAAACTCGTGGATGGACGTTCAACATTGAGGAAGGCATAACGCTACTACCTGATGTTTACTCCAACCTGATTGTATACAGTGACGACTATTTATCCCTAATGt                                              <  1:489720/301‑1 (MQ=255)`  
                                                                                                                                                                                                                                                             `cTTGAGGGGTTTTTTGCTGAAAGGAGGAACTATATGCGCTCATGCGATATGAACGTTGAGACTGCCGCTGAGTTATCAGCTGTGAACGACATTCTGGCGTCTATCGGTGAACCTCCGGTATCAACGCTGGAAGGTGACGCTAACGCAGATGCAGCGAACTCTCGGTGTATTCTCAACAAGATTAACCGACAGATTCCATCTCGTGGATGGACGTTCAACATTGAGGAAGGCATAACGCTACTACCTGATGTTTACTCCAACCTGATTGTATACAGTGACGACTATTTATCCCtgtctctta                                           >  1:538529/1‑293 (MQ=255)`  
                                                                                                                                                                                                                                                              `cgtgggctcggagatgtgtataagagacagatatGCGCTCATGCGATATGAACGTTGAGACTGCCGCTGAGTTATCAGCTGTGAACGACATTCTGGCGTCTATCGGTGAACCTCCGGTATCAACGCTGGAAGGTGACGCTAACGCAGATGCAGCGAACGCTCGGCGTATTCTCAACAAGATTAACCGACAGATTCAATCTCGTGGATGGACGTTCAACATTGAGGAAGGCATAACGCTACTACCTGATGTTTACTCCAACCTGATTGTACACAGTGACGACTATTTATCCCTAATGTCTAc                                          <  1:730826/271‑1 (MQ=255)`  
                                                                                                                                                                                                                                                               `tGAGGGGTTTTTTGATGAAAGGAGGAACTATATGCGCTCATGCGATATGAACGTTGAGACTGCCGCTGAGTTATCAGCTGTGAACGACATTCTGGCGTCTATCGGTGAACCTCCGGTATCAACGCTGGAAGGTGACGCTAACGCAGATGCAGCGAACACTCGGCGTATTCTCAACAAGATTAACCGACAGATTCAATCTCGTGGATGGACGTTCAACATTGAGGAAGGCATAACGCTACTACCTGATGTTTACTCCAACCTGATTGTATACAGTGACGACTATTTATCCCTGATGTCTACt                                         <  1:563321/301‑1 (MQ=255)`  
                                                                                                                                                                                                                                                                 `gtgggctcggagatgtgtataagagacagatGCGCTCATGCGATATGAACGTTGAGACTGCCGCTGAGTTATAAGCTGTGAACGACATTCTGGCGTCTATCGGTGAACCTCCGGTATCAACGCTGGAAGGTGACGCTAACGCAGATGCAGCGAACGCTCGGCGTATTCTCAACAAGATTAACCGACAGATTCAATCTCGTGGATGGACGTTCAACATTGAGGAAGGCATAACGCTACTACCTGATGTTTACTCCAACCTGATTGTATACAGTGACGACTATTTATCCCTAATGTCTACTTc                                       <  1:125740/272‑1 (MQ=255)`  
                                                                                                                                                                                                                                                                 `aGGGGTTTTTTGCTGAAAGGAGGAACTATATGCGCTCATGCGATATGAACGTTGAGACTGCCGCTTAGTTATCAGCTGTGAACGACATTCTGGCGTCTATCGGAGAACCTCCGGTATCAACGCTGGAAGGTGACGCTAACGCAGATGCAGCGAACGCTCGGTGTATTCTCAACAAGATTAACCGACAGATTCAATCTCGTGGATGGACGTTCAACATTGAGGAAGGCATAACGCTACTACCTGATGTTTACTCCAACCTGATTGTATACAGTGACGACTATTTATCCCTAATGTCTACTTc                                       <  1:665226/301‑1 (MQ=255)`  
                                                                                                                                                                                                                                                                      `ttttttGCTGAAAGGAGGAACTATATGCGCTCATGCGATATGAACGTTGAGACTGCCGCTGAGTTATCAGCTGTGAACGACATTCTGGCGTCTATCGGTGAACCTCCGGTATCAACGCTGGAAGGTGACGCTAACGCAGATGCAGCGAACGCTCGGCGTATTCTCAACAAGATTAACCGACAGATTCAATCTCGTGGATGGACGTTCAACATTGAGGAAGGCATAACGCTACTACCTGATGTTTACTCCAACCTGATTGTATACAGTGACGACTATTTATCCCTAATGTCTACTTCCGGTc                                  <  1:409695/301‑1 (MQ=255)`  
                                                                                                                                                                                                                                                                          `gatgtgtataagagacagatatGCGCTCATGCGATATGAACGTTGAGACTGCCACTGAGTTATCAGCTGTGAACGACATTCTGGCGTCTATCGGTGAACCTCCGGTATCAACGCTGGAAGGTGACGCTAACGCAGATGCAGCGAACGCTCGGCGTATTCTCAACAAGATTAACCGACAGATTCAATCTCGTGGATGGACGTTCAACATTGAGGAAGGCATAACGCTACTACCTGATGTTTACTCCAACCTGATTGTATACAGTGACGACTATTTATCCCTAATGTCTACTTCCGGTCAATc                              <  1:149212/283‑1 (MQ=255)`  
                                                                                                                                                                                                                                                                                 `aaGGAGGAACTATATGCGCTCATGCGATATGAACGTTGAGACTGCCGCTGAGTTATCAGCTGTGAACGACATTCTGGCGTCTATCGGTGAACCTCCGGTATCAACGCTGGAAGGTGACGCTAACGCAGATGCAGCGAACGCTCGGCGTATTATCAACAAGATTAACCGACAGATTCAATCTCGTGGATGGACGTTCAACATTGAGGAAGGCATAACGCTACTACCTGATGTTTACTCCAACCTGATTGTATACAGTGACGACTATTTATcccctgtctcttatacacatctccgagcccac                       >  1:453883/1‑272 (MQ=255)`  
                                                                                                                                                                                                                                                                                    `gaggaACTATATGCGCTCATGCGATATGAACGTTGAGACTGCCGCTGAGTTATCAGCTGTGAACGACATTCTGGCGTCTATCGGTGAACCTCCGGTATCAACGCTGGAAGGTGACGCTAACGCAGATGCAGCGAACGCTCGGCGTATTCTCAACAAGATTAACCGACAGATTCAATCTCGTGGATGGACGTTCAACATTGAGGAAGGCATAACGCTACTACCTGATGTTTACTCCAACCTGATTGTATACAGTGACGACTATTTATCCCTAATGTCTACTTCCGGTCAATCCATCTACGtt                    >  1:820464/1‑301 (MQ=255)`  
                                                                                                                                                                                                                                                                                      `ggaACTATATGCGCTCATGCGATATGAACGTTGAGACTGCCGCTGAGTTATCAGCTGTGAACGACATTCTGGCGTCTATCGGTGAACCTCCGGTATCAACGCTGGAAGGTGACGCTAACGCAGATGCAGCGAACGCTCGGCGTATTCTCAACAAGATTAACCGACAGATTCAATCTCGTGGATGGACGTTCAACATTGAGGAAGGCATAACGCTACTCCCTGATGTTTACTCCAACCTGATTGTATACAGTGACGACTATTTATCCCTAATGTCTACTTCCGGTCAATCCATCTACGTTaa                  >  1:700242/1‑301 (MQ=255)`  
                                                                                                                                                                                                                                                                                       `gaACTATATGCGCTCATGCGATATGAACGTTGAGACTGCCGCTGAGTTATCAGCTGTGAACGACATTCTGGCGTCTATCGGTGAACCTCCGGTATCAACGCTGGAAGGTGACGCTAACGCAGATGCAGCGAACGCTCGGCGTATTCTCAACAAGATTAACCGACAGATTCAATCTCGTGGATGGACGTTCAACATTGAGGAAGGCATAACGCTACTACCTGATGTTTACTCCAACCTGATTGTATACAGTGACGACTATTTATCCCTAATGTCTACTTCCGGTCAATCCATCTACGTTAAc                 >  1:47072/1‑301 (MQ=255)`  
                                                                                                                                                                                                                                                                                        `aaCTATATGCGCTCATGCGATATGAACGTTGAGACTGCCGCTGAGTTATCAGCTGTGAACGACATTCTGGCGTCTATCGGTGAACCTCCGGTATCAACGCTGGAAGGTGACGCTAACGCAGATGCAGCGAACGCTCGGCGTATTCTCAACAAGATTAACCGACAGATTCAATCTCGTGGATGGACGTTCAACATTGAGGAAGGCATAACGCTACTACCTGATGTTTACTCCAACCTGATTGTATACAGTGACGACTATTTATCCCTAATGTCTACTTCCTGTctcttatacacatctccga                >  1:224097/1‑283 (MQ=255)`  
                                                                                                                                                                                                                                                                                        `aaCTATATGCGCTCATGCGATATGAACGTTGAGACTGCCGCTGAGTTATCAGCTGTGAACGACATTCTGGCGTCTATCGGTGAACCTCCGGTATCAACGCTGGAAGGTGACGCTAACGCAGATGCAGCGAACGCTCGGCGTATTCTCAACAAGATTAACCGACAGATTCAATCTCGTGGATGGACGTTCAACATTGAGGAAGGCATAACGCTACTACCCGATGTTTACTCCAACCTGATTGTATACAGTGACGACTATTTATCCCTAATGTCTACTTCCTGTctcttatacacatctccga                >  1:486280/1‑283 (MQ=255)`  
                                                                                                                                                                                                                                                                                          `cTATATGCGCTCATGCGATATGAACGTTGAGACTGCCGCTGAGTTATCAGCTGTTAACGACATTCTGGCGTCTATCGGTGAACCTCTGGTATCAACGCTGGAAGGTGACGCTAACGCAGATGCAGCGAACGCTCGGCGTATTCTCAGCAAGATTAACCGACAGCTTCAATCTCGTGGATGGACGTTCAACATTGAGGAAGGCATAACGCTACTACCTGATGTTTACTCAAACCTGATTGTATACAGTGACGACTAGGTATCCCTAATGTCTACTTCCGGtgctgtctcttatacacatctc              >  1:64477/1‑280 (MQ=255)`  
                                                                                                                                                                                                                                                                                          `cTATATGCGCTCATGCGATATGAACGTTGAGACTGCCGCTGAGTTATCAGCTGTGAACGACATTCTGGCGTCTATCGGTGAACCTCCGGTATCAACGCTGGAAGGTGACGCTAACGCAGATGCAGCGAACGCTCGGCGTATTCTCAACAAGATTAACCGACAGATTCAATCTCGTGGATGGACGTTCAACATTGAGGAAGGCATAACGCTACTACCTGATGTTTACTCCAACCTGATTGTATACAGTGACGACTATTTATCCCTAATGTCTACTTCCGGTCAATCCATCTACGTTAACTGa              >  1:677337/1‑301 (MQ=255)`  
                                                                                                                                                                                                                                                                                          `cTATATGCGCTCATGCGATATGAACGTTGAGACTGCCGCTGAGTTATCAGCTGTGAACGACATTCTGGCGTCTATCGGTGAACCTCCGGTATCAACGCTGGAAGGTGACGCTAACGCAGATGCAGCGAACGCTCGGCGTATTCTCAACAAGATTAACCGACAGATTCAATCTCGTGGATGGACGTTCAACATTGAGGAAGGCATAACGCTACTACCTGATGTTTACTCCAACCTGATTGTATACAGTGACGACTATTTATCCCTAATGTCTACTTCCGGTCAATCCATCTACGTTAACCGa              >  1:240961/1‑301 (MQ=255)`  
                                                                                                                                                                                                                                                                                            `atatGCGCTCATGCGATATGAACGTTGAGACTGCCGCTGAGTTATCAGCTGTGAACGACATTCTGGCGTCTATCGGTGAACCTCCGGTATCAACGCTGGAAGGTGACGCTAACGCAGATGCAGCGAACGCTCGGCGTATTCTCAACAAGATTAACCGACAGATTCAATCTCGTGGATGGACGTTCAACATTGAGGAAGGCATAACGCTACTACCTGATGTTTACTCCAACCTGATTGTATACAGTGACGACTATTTATCCCTAATGTCTACTTCCGGTCAATCCATCTACGtctgtctctt            >  1:203743/1‑292 (MQ=255)`  
                                                                                                                                                                                                                                                                                                   `cTCATGCGATATGAACGTTGAGACTGCCGCTGAGTTATCAGCTGTGAACGACATTCTTGCGTCTATCGGTGAACCTCCGGTATCAACGCTGGAAGGTGACGCTAACGCAGATGCAGCGAACGCTCGGCGTATTCTCAACAAGATTAACCGACAGATTCAATCTCGTGTATGGACGTT‑AACATTGAGGAAGGCATAACGCTACTACCTGATGTTTACTCCAACCTGATTGTATACAGTGACGACTATTTATCCCTAATGTCTACTTCCGGTCAATCCATCTACGTTAACCGAGGTGGCTAtt    >  1:578841/1‑300 (MQ=255)`  
                                                                                                                                                                                                                                                                                                     `cATGCGATATGAACTTTGAGACTGCCGCTGAGTTATCAGCTGTGAACGACATTCTGGCGTCTATCGGTGAACCTCCGGTATCAACGCTGGAAGGTGACGCTAACGCAGATGCAGCGAACGCTCGGCGTATTCTCAACAAGATTAACCGACAGATTCAATCTCGTGGATGGACGTTCAACATTGAGGAAGGCATAACGCTACTACCTGATGTTTACTCCAACCTGATTGTATACAGTGACGACTATTTATCCCTAATGTCTACTTCCGGTCAATCCATCTACGTTAACCGAGGTGGCTAtgt   >  1:160313/1‑301 (MQ=255)`  
                                                                                                                                                                                                                                                                                                     `cATGCGATATGAACGTTGAGACTGCCGCTGAGTTATCAGCTGTGAACGACATTCTGGCGTCTTTCGGTGAACCTCCGGTATCAACGCTGTCAGGTGACGCTAACGCAGATGCAGCGAACGCTCGGCGTATTCTCAACAAGATTAACCGACAGATTCAATCTCGTTGATGGACGTTCCACATTGAGGAAGGCATAACGCTACTACCTGATGTTTACTCCAACCTTA‑TGTATACAGTGACGACTATTTATACCAAATGTCTACTTCCGTCCAATCCATCTACGTTAACCGCGGTGGCTAtgtt  >  1:802909/1‑300 (MQ=255)`  
                                                                                                                                                                                                                                                                                                     `cATGCGATATGAACGTTGAGACTGCCGCTGAGTTATCAGCTGTGAACGACATTCTGGCGTCTATCGGTGAACCTCCGGTATCAACGCTGGAAGGTGACGCTAACGCAGATGCAGCGAACGCTCGGCGTATTCTCAACAAGATTAACCGACAGATTCAATCTCGTGGATGGACGTTCAACATTGAGGAAGGCATAACGCTACTACCTGATGTTTACTCCAACCTGATTGTATACAGTGACGACTATTTATCCCTAATGTCTACTTCCGGTCAATCCATCTACGTTAACCGAGGTGGCTAtgt   >  1:344602/1‑301 (MQ=255)`  

                                                                                                                                                                                                                                                                                                        `|`                                                                                                                                                                                                                                                                                                            
`GCAATGGGCCACGGTGGTCTTCGCCCAGAAGCTGCTGGTGCAGTGGTTTTCAAAGTGGAGTAATGCTGGGGGTGGCCTCAACGGTCGCTGCTAGTCCCGAAGAGGCGAGTGTTACTTCAACAGAAGAAACCTTAACGCCAGCACAGGAGGCCGCACGCACCCGCGCTGCTAACAAAGCCCGAAAGGAAGCTGAGTTGGCTGCTGCCACCGCTGAGCAATAACTAGCATAACCCCTTGGGGCCTCTAAACGGGTCTTGAGGGGTTTTTTGCTGAAAGGAGGAACTATATGCGCTCATACGATATGAACGTTGAGACTGCCGCTGAGTTATCAGCTGTGAACGACATTCTGGCGTCTATCGGTGAACCTCCGGTATCAACGCTGGAAGGTGACGCTAACGCAGATGCAGCGAACGCTCGGCGTATTCTCAACAAGATTAACCGACAGATTCAATCTCGTGGATGGACGTTCAACATTGAGGAAGGCATAACGCTACTACCTGATGTTTACTCCAACCTGATTGTATACAGTGACGACTATTTATCCCTAATGTCTACTTCCGGTCAATCCATCTACGTTAACCGAGGTGGCTATGTG  >  NC_001604.1/23942‑24536`

  

Alignment Legend

Aligned base mismatch/match (shaded by quality score): `ATCG/ATCG` `< 3 ≤` `ATCG/ATCG` `< 15 ≤` `ATCG/ATCG` `< 26 ≤` `ATCG/ATCG` `< 36 ≤` `ATCG/ATCG` `< 38 ≤` `ATCG/ATCG`

Unaligned base: `atcg`    Masked matching base: `atcg`    Alignment gap: `‑`    Deleted base: `‑`
